# BrisSynBio: a BBSRC/EPSRC-funded Synthetic Biology Research Centre

**DOI:** 10.1042/BST20160004

**Published:** 2016-06-09

**Authors:** Kathleen R. Sedgley, Paul R. Race, Derek N. Woolfson

**Affiliations:** * BrisSynBio, Life Sciences Building, Tyndall Avenue, Bristol BS8 1TQ, U.K.; † School of Biochemistry, University of Bristol, Bristol BS8 1TD, Avon, U.K.; ‡ School of Chemistry, University of Bristol, Bristol BS8 1TS, Avon, U.K.

**Keywords:** biotechnology, molecular modelling, protein design, protein engineering, synthetic biology

## Abstract

BrisSynBio is the Bristol-based Biotechnology and Biological Sciences Research Council (BBSRC)/Engineering and Physical Sciences Research Council (EPSRC)-funded Synthetic Biology Research Centre. It is one of six such Centres in the U.K. BrisSynBio's emphasis is on rational and predictive bimolecular modelling, design and engineering in the context of synthetic biology. It trains the next generation of synthetic biologists in these approaches, to facilitate translation of fundamental synthetic biology research to industry and the clinic, and to do this within an innovative and responsible research framework.

BrisSynBio is supported by £13.6 million from Biotechnology and Biological Sciences Research Council (BBSRC)/Engineering and Physical Sciences Research Council (EPSRC), which includes a £3.3 million investment in new state-of-the-art equipment and computing, and significant additional University of Bristol support. BrisSynBio is led by the University of Bristol in partnership with the University of the West of England. It is a multidisciplinary Centre distributed across four Faculties, with an administrative hub in University of Bristol's new £56 million Life Sciences Building (Figure 1). BrisSynBio partners include: SynBiCITE, the Imperial College-led synthetic biology industrial translation engine; Oxford and Warwick to deliver the EPSRC/BBSRC-funded Centre for Doctoral Training in Synthetic Biology; and the EU-wide SYNENERGENE project exploring public engagement in synthetic biology.

**Figure 1:**
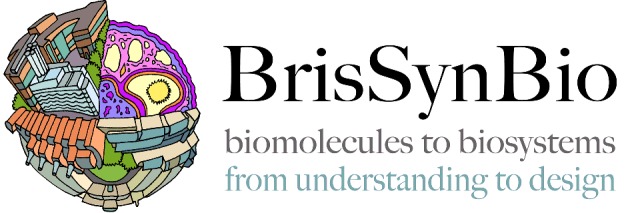
BrisSynBio illustration by Andy Council, 2014 This image was created by Andy Council in collaboration with BrisSynBio in 2014. Andy is a Bristol-based artist and illustrator. His style is instantly recognizable in and around Bristol: he ‘creates composite beasts made up of architectural landmarks and other recognizable elements’; and some of his work is visible in the streets of Bristol and on display at the M Shed in the city. The illustration invites the viewer to take a peek inside a cell through a window cut into the cell wall, with the latter created from illustrations of the buildings at the University of Bristol most closely associated with BrisSynBio.

BrisSynBio's Director, Professor Dek Woolfson, is one of the U.K.'s leading advocates for advancing and broadening synthetic biology approaches to include biomolecular design and engineering [1]. He has published influential papers in these areas [2–4], well-cited reviews [1] and commentaries [5]. Woolfson's research is at the interface between the physical and biological sciences, and specifically in computational and experimental aspects of protein design and its application to synthetic biology. He is supported by the Centre's Co-Director, Dr Paul Race, whose work focuses on the exploitation and manipulation of enzyme complexes, pathways and networks, en route to developing new tools, technologies and products for red, green and white biotechnology. Race has co-authored papers in leading international journals [6–8] and has a strong track record of industrial engagement.

BrisSynBio's scientific focus is on the rational design and engineering of nucleic acids, lipids, peptides and proteins as structural, enzymatic and regulatory components in new biological and bio-inspired systems. BrisSynBio is organized into three research Strands:

**Strand 1–Enzyme Cascades and Cell Factories**: This strand focuses on the isolation, characterization and scalable production of ‘new-to-science’ natural product-based therapeutics and agrochemicals. A major emphasis is polyketides, the most structurally and functionally diverse family of bioactive natural products known. These molecules represent challenging, frequently intractable targets for synthetic organic chemistry, and as such biosynthesis offers the only generally applicable route to efficient production. Exemplar projects include: engineering *Trans*-acyltransferase (AT) polyketide synthase (PKS) antibiotic gene clusters to deliver bioactive compounds, led by Willis [9]; scalable biosynthetic platforms for polyketide production in *Escherichia coli,* led by Race [6–8]; and molecular membrane engineering for nanoreactor bionetworks, led by Collinson [10].

**Strand 2–Self-assembled Systems and Minimal Cells**: This strand explores using and combining stripped-down biological components and machinery to build virus- and cell-like micro-compartments. It involves the systematic inclusion and networking of engineered biomolecular and cellular components, and gene circuitry to establish biomimetic operations and modules. Achieving this vision depends critically on the construction and design of self-assembled systems, novel representations of synthetic cellularity and re-engineering of minimal cells. Exemplar projects include: developing hybrid peptide-virus nanoparticles for immunology and cell delivery, led by Woolfson [1–4]; design and construction of solar energy transducing oxidoreductase cascades within metabolic protocells, led by Mann [11]; and engineering red blood cells as drug delivery agents and bioreactors, led by Toye [12].

**Strand 3–Programming Complexity in Natural Systems**: Strand 3 integrates modelling, cell biology and imaging to address the predictable reprogramming and control of single cells, cell populations and cells in multicellular organisms. Applications are envisaged for whole-cell biosensors for the detection, analysis and remediation of aromatic pollutants; the use of engineered cells to control cell populations; and the development of wheat strains with the potential to revolutionize wheat breeding for agriculturally important traits. Exemplar projects include: design and implementation of orthogonal components for transcriptional logic gates, led by Savery [13]; harnessing synthetic oscillators, led by di Bernardo [14]; and a synthetic pathway for recombination in wheat, led by Edwards [15]. The first BrisSynBio Fellow in Synthetic Biology, Gorochowski, has joined this Strand working on *tools for the rational design of synthetic gene circuits* [16].

The Strands are supported by three cross-cutting Themes in: *Design and Characterization of Biomolecular Components*; *Engineering and Modelling across Scales*; and *Public Engagement and Responsible Research and Innovation.* For the second of these, three computational scientists are employed to cover theoretical aspects and computational modelling and simulation. Sophisticated models can help predict the outcome of experiments, and hence inform which experiments are most likely to provide useful outcomes. Additionally, they offer powerful methods for interpreting experimental results. BrisSynBio has built upon existing close relationships between modelling and experimental groups by providing an interface across the scales of modelling that are addressed by different research groups at Bristol so that experimental researchers can readily collaborate with the appropriate group for the scale of problem they are addressing: atomistic molecular modelling and simulation, led by Sessions and co-workers [2,3,17]; modelling catalytic mechanisms, led by Mulholland [18]; and coarse-grained modelling, led by Liverpool [19].

In Theme 3, Kent [20] leads the project exploring *fundamental problems, ethics and responsible innovation in the design and engineering of synthetic-biological systems*. A philosopher works alongside BrisSynBio researchers, examining what ‘responsible innovation’ and ‘ethical science’ mean in practice, as well as fundamental ethical and philosophical questions raised by BrisSynBio's research and synthetic biology activities more broadly. As part of this aspect of BrisSynBio, the Centre and its researchers are proactively involved in a range of public engagement, from traditional science cafes and visits to local schools, to innovations such as Question Time-style panel discussions and supporting a show at the 2015 Edinburgh Fringe [21].

All of these research activities are underpinned by major new facilities supported through the Centre grant for: high-throughput molecular biology; NMR spectroscopy and peptide synthesis; high-performance computing; microfluidics; FACS; and advanced microscopy and imaging. And to help pave the way to applications, BrisSynBio's current industrial partners include Bruker, GSK, Syngenta and UCB, through which BrisSynBio aims to realize translational outcomes in the areas of fine and specialty chemicals, medicines and healthcare, and wheat breading.

## Abbreviations

AT: acyltransferase
BBSRC: Biotechnology and Biological Sciences Research Council
EPSRC: Engineering and Physical Sciences Research Council
PKS: polyketide synthase
